# Effect of COP1 in Promoting the Tumorigenesis of Gastric Cancer by Down-Regulation of CDH18 via PI3K/AKT Signal Pathway

**DOI:** 10.1155/2023/5617875

**Published:** 2023-03-28

**Authors:** Benhuo Zhao, Jiaojiao Wu, Xiuli Cha, Guangtong Mao, Hengliang Shi, Sujuan Fei, Bei Miao

**Affiliations:** ^1^Department of Gastroenterology, The Affiliated Hospital of Yangzhou Medical University, 368 Hanjiang Middle Road, Yangzhou, 225100 Jiangsu Province, China; ^2^Department of Gastroenterology, The Affiliated Hospital of Xuzhou Medical University, 99 West Huaihai Road, Xuzhou, 221002 Jiangsu, China; ^3^Institute of Digestive Diseases, Xuzhou Medical University, 84 West Huaihai Road, Xuzhou, 221002 Jiangsu, China; ^4^Department of Pathology, Xinyi People's Hospital, 16 Renmin Road, Xinyi, 221400 Jiangsu, China; ^5^Central Laboratory, The Affiliated Hospital of Xuzhou Medical University, 99 West Huaihai Road, Xuzhou, 221002 Jiangsu, China

## Abstract

In recent years, the involvement of E3 ubiquitin ligase constitutive photomorphogenesis 1 (COP1) in the tumorigenesis of gastric cancer (GC) has been elucidated. However, the exact underlying mechanism remains to be clarified. In the present study, the expression profiles of COP1 in GC were derived from the Gene Expression Omnibus (GEO) and the Cancer Genome Atlas (TCGA) databases, followed by verification via immunohistochemical staining (IHC), Western blotting (WB), and quantitative real-time polymerase chain reaction (qRT-PCR) reaction assays on clinical samples. In vitro, the gain- and loss-of-function experiments of COP1 protein were conducted to explore its role in GC cell lines HGC-27 and SGC-7901. Furthermore, we screened the interaction protein of COP1 by yeast two-hybrid experiment and verified their combination by co-immunoprecipitation (co-IP). We preliminary explored the possible underlying mechanisms of COP1 protein in GC cell lines via WB. COP1 was upregulated in GC tissues compared with the corresponding non-carcinoma tissues. In vitro, the upregulation of COP1 protein promoted the proliferation and migration of GC cells. The yeast two-hybrid experiment and co-IP indicated that Cadherin 18 (CDH18) could constitute a complex with COP1. Moreover, cells with COP1 over-expression showed low levels of CDH18 expression, with the intracellular PI3K/AKT pathway activated and the malignancy of GC cell lines enhanced. Our findings demonstrated that COP1 promoted the GC tumorigenesis by downregulated CDH18 with the involvement of PI3K/AKT signaling pathway in cell lines, suggesting the potential of COP1 as a prognostic biomarker and therapeutic target for GC.

## 1. Introduction

Gastric cancer (GC) is the fourth contributor of cancer-related death in underdeveloped and developing countries [1]. Surgical resection and endoscopy therapy are the curative treatment currently available for patients diagnosed with GC in early stage. However, there is a low survival rate in the majority of patients with advanced GC worldwide even by adjuvant therapies with chemotherapy [2] and chemoradiation [3]. In the perioperative period, immunotherapy combined with targeted therapy may be reportedly promising in enhancing the efficacy for advanced GC [4, 5]. The quest for ideal biomarkers of GC for the targeted therapy is highlighted.

Ubiquitin–proteasome system (UPS)-mediated ubiquitination is an approach of post-translational modification of proteins. Intracellular biological processes such as cell proliferation, cell-cycle progression, transcription, and apoptosis are regulated by ubiquitination [6]. The protein ubiquitination requires the participation of ubiquitin-activating enzyme E1, ubiquitin-binding enzyme E2, and ubiquitin ligase E3 [7]. Emerging studies demonstrate that constitutive photomorphogenesis 1 (COP1), as an E3 ligase, is abnormally expressed in a wealth of tumor types, leading to the dysregulation of proteins related to tumor suppressor genes p53, p27, 14-3-3*σ* [8–10] as well as oncogenes c-Jun, signal transducer and activator of transcription 3 (STAT3), E-twenty-six (ETS) family members [11–13], etc. The knowledge highlights the involvement of COP1 in tumorigenesis [14, 15]. Moreover, COP1 plays a pivotal role in the activation of PI3K/AKT pathway in tumor cells [16–18]. However, the role of COP1 in the development of GC still remains controversial [19, 20]. Therefore, more evidence should be required to unmask the exact impact of COP1 dysregulation in GC cells and the underlying mechanisms.

## 2. Materials and Methods

### 2.1. Bioinformatics Analysis

The mRNA expression data of COP1 was obtained from the Gene Expression Omnibus (GEO) database (GSE27342 dataset, 80 cases of GC tissues, and their corresponding adjacent tissues) and the Cancer Genome Atlas (TCGA) database (415 cases of GC tissues and 34 cases of adjacent tissues). Furthermore, the differential expressions of COP1 in GC tissues were statistically analyzed by the Graphpad Prism 8.2 and SPSS 22.0 software.

### 2.2. Tissue Samples

The present study enrolled 34 samples of patients diagnosed with GC who received subtotal gastrectomy and hospitalized in the Department of Gastrointestinal Surgery of the Affiliated Hospital of Xuzhou Medical University between March 2020 and February 2021. The inclusion criteria were patients who had not received anti-tumor therapy before surgery and postoperative pathology confirmed to be gastric adenocarcinoma. Each GC tissue and the adjacent noncancerous gastric tissue (as a pair) were processed immediately following the resection: 34 pairs of samples were finally obtained, 12 pairs were fixed in formalin for immunohistochemical staining (IHC), and 22 pairs were frozen in liquid nitrogen (18 for Western blotting [WB], 4 for the construction of cDNA library). This study was approved by the Ethics Committee of the Affiliated Hospital of Xuzhou Medical University.

### 2.3. Immunohistochemical Staining

The formalin-fixed tissues were paraffin-embedded, sliced into sections at 4 *μ*m thickness. IHC staining was performed with the use of the COP1 primary antibody (1 : 200, Affinity Sciences, JiangSu, China) and the Rabbit Two-step immunohistochemical kit (ZSGB-Bio. Co, Ltd., Beijing, China) according to the instructions. The slices were dyed via 3,3′-diaminobenzidine-tetrahydrochloride-dihydrate (DAB) and visualized by the positive placement microscope (Olympus, Tokyo, Japan). Each tissue image was captured by OCULAR software (Auckland, New Zealand) with five random nonoverlapping fields (under 20× light microscope) digitalized into mean values of integrated optical density (IOD) via Image Pro-Plus 6.0 software (Media Cybernetics, Silver Spring, MD, USA).

### 2.4. Cell Culture

Human GC cell lines (HGC-27 and SGC-7901) and the human embryonic kidney (HEK) 293T cell line were purchased from the Cell Bank of the Chinese Academy of Sciences (Shanghai, China). GC cells were cultured in RPMI-1640 medium (Hyclone, USA) with 10% fetal bovine serum (Gibco, USA). HEK 293T cells were cultured with Dulbecco's Modified Eagle medium (DMEM) (Hyclone, USA) with 10% fetal bovine serum. Cells were maintained in a humidified cell incubator at 37°C with an atmosphere of 5% CO_2_.

### 2.5. Lentivirus Infection and Cell Transient Transfection

The endogenous EF1a-GFP vector for COP1 over-expression, lentivirus-containing short hairpin RNA (shRNA) for COP1 silencing, and their corresponding control vectors were purchased from GenePharma (Shanghai, China). With the aid of Polybrene (6 *μ*g/mL, Beyotime, China), the GC cell lines were infected for 48 hours, followed by puromycin perfusion (6 *μ*g/mL, Beyotime, China) for 1 week. The establishment of stable COP1 over-expressing and silencing cell lines was verified by WB. The CDH18 si-RNA and its negative control (NC) oligonucleotide were also obtained from GenePharma, CDH18 over-expression plasmid and its vector control plasmid were purchased from YouBio (Hunan, China). CDH18 plasmids and RNAs were transient transfected into GC cell lines with the usage of Lipofectamine 8,000 reagent (Beyotime, China) according to the manufacturer's instructions.

### 2.6. CCK-8, EdU, and Colony Formation Assays

Cell proliferation was assessed using the CCK8 kit (APExBio, USA) according to the instructions. 5,000 cells of each group were seeded, 5 repetitions, quintuplicated, then incubated in 96-well plates at 37°C. Then the original medium in each well was discarded and replaced with fresh complete medium containing 10% CCK-8 regent for 2-hour incubation at 37°C at the same time in a total of four consecutive days. The absorbance at 450 nm of each well was determined with the microplate reader (Spark, Sweden). 5-ethynyl-2′-deoxyuridine (EdU) experiment was performed according to the BeyoClick™ EDU-555 kit (Beyotime, China) instructions. 10,000 cells were seeded with five repetitions and cultured into a 96-well plate at 37°C. 200 *μ*L of medium with 10 mM EdU solution was added into each well for further incubation of 2 hours. Then mediums in each well were discarded, and cells were fixed with 4% paraformaldehyde and permeabilized with 0.3% Triton X-100 for 15 minutes at room temperature (r/t). After that, the solutions were discarded and replaced with the click addictive solution (50 *μ*L per well) for 30 minutes of incubation illuminated. Finally, the cells were illuminated for 5 minutes of incubation with Hoechst 33342 (10 *μ*g/ml), rinsed by phosphate buffer solution (PBS), and captured under the inverted fluorescence microscopy (IX71; Olympus, Japan). For colony formation assay, 1,000 cells were cultured into a 6 cm Petri dish with 6 ml of complete medium for 1 week. Then the cells were rinsed twice with PBS, fixed by 4% paraformaldehyde, and stained with crystal violet solution (VicMed Life Sciences, China). At last, the cells were rinsed twice with ddH_2_O and dried, captured by camera and the numbers of colonies were counted.

### 2.7. Cell Migration and Invasion Assays

Migration and invasion assays were performed in 24-well plate, with a 8 *μ*m pore size porous membrane filter (Corning, New York, USA) inserted, with or without pre-coated diluted Matrigel (Corning, New York, USA). 15,000 cells were resuspended with serum-free medium and seeded into the upper chamber, the medium containing 10% fetal bovine serum (FBS) was added into the bottom chamber. Followed by the incubation for 16 hours at 37°C, the cells of the inner side of the upper chamber were gently wiped with a cotton swab, and the outside membranes of the upper chamber were rinsed once with PBS and fixed with 4% formaldehyde for 10 minutes. Thereafter, the cells of outside membranes were stained by crystal violet solution and captured under the microscope with the cell population numbers in five random fields calculated.

### 2.8. WB Assay

Following the preparation of the total protein, each sample of identical quantity was added into the individual lanes. The protein samples were separated by 8% sodium dodecyl sulfate (SDS)-polyacrylamide gel electrophoresis and transferred onto polyvinylidene fluoride (PVDF) membranes (Millipore, USA). After blockaded with 3% Bovine Serum Albumin (BSA) in Tris-buffered saline Tween-20 buffer (Solarbio Sciences, China), the membranes were incubated with primary antibodies at 4°C overnight, respectively. The dilution concentrations of the reagents were as follows: COP1, CDH18, AKT, and p-AKT antibodies were obtained from Affinity Sciences (Jiangsu, China), and the dilution concentration was 1 : 500; phosphorylated mammalian target of rapamycin (p-mTOR) antibody was purchased from Abcam (Cambridge, UK), and the dilution concentration was 1 : 1,000; and *β*-actin primary antibody was obtained from Protein-tech (Wuhan, China), at the dilution of 1 : 5,000. Horse Radish Peroxidase (HRP)-conjugated secondary antibodies (diluted 1 : 5,000 with Tris-buffered saline with Tween-20 (TBST) were used to detect the binding of primary antibodies. Bands were chemically illuminated with the use of enhanced chemiluminescence (ECL) reagent (Affinity Sciences, China) and scanned with chemiluminescence imaging analysis system (Tanon, China). Bands were analyzed using ImageJ software.

### 2.9. Co-Immunoprecipitation Assay

The HEK 293T cells were seed into a 6 cm plate, 5 *μ*g COP1 over-expression plasmid was transient transfected after the cells adhered, about 42 hours after transfection, cells were processed with 10 *μ*M protease inhibitor cocktail MG-132 (Med Chem Express, Shanghai, China) for 6 hours. Then cells were collected and lysed with NP-40 lysis buffer (Beyotime, Nanjing, China). 40 *μ*g of total protein was prepared as the input group, and the remaining proteins were prepared into a protein system with the concentration of 1 mg/mL and the volume of 1 mL into two tubes. The anti-COP1 antibody (10 *μ*g) and the immunoglobulin G (IgG) antibody (10 *μ*g) were respectively added into the total protein, then slowly agitated overnight at 4°C. The protein A/G agarose (Beyotime, Nanjing, China) was added and incubated for 4 hours at 4°C, followed by triplicate lavage with pre-cooled TBS solution, samples were detected by WB.

### 2.10. quantitative real-time polymerase chain reaction

Trizol reagent (Thermo Fisher Scientific, USA) was adopted for extraction of total RNA from GC and adjacent gastric tissues according to the manufacturer's instructions, with the reverse transcription process performed via the PrimeScript RT Master Mix (Tiangen, Beijing, China). Thereafter, the expression of COP1 mRNA of tissues was examined using SYBR Green qPCR Master Mix (MCE, Shanghai, China), with 40 cycles of 95°C for 10 seconds and 60°C for 30 seconds. The primers were purchased from Sangon Biotech, China, and the sequences were as follows:

COP1-F, 5′-CTGTTTGGGAGGTCGGGTAAATGG-3′

COP1-R, 5′-AGTGGTGTGAGTGAGAGGCTGAG-3′.

### 2.11. Statistical Analysis

The SPSS 22.0 and Graphpad Prism 8.2 tools were used for statistical analysis, and the data was expressed as the mean ± SD. Student's *t*-test was adopted to test for differences in the comparison of each two groups, and one-way analysis of variance (ANOVA) was used to determine differences among at least three groups, follow by Tukey's *post hoc* test. The normality test was conducted before Student's *t*-test or ANOVA test performed. Difference at *p* < 0.05 was considered as statistically significant.

## 3. Results and Discussion

### 3.1. COP1 Was Upregulated in GC Tissues

**Figure 1:**
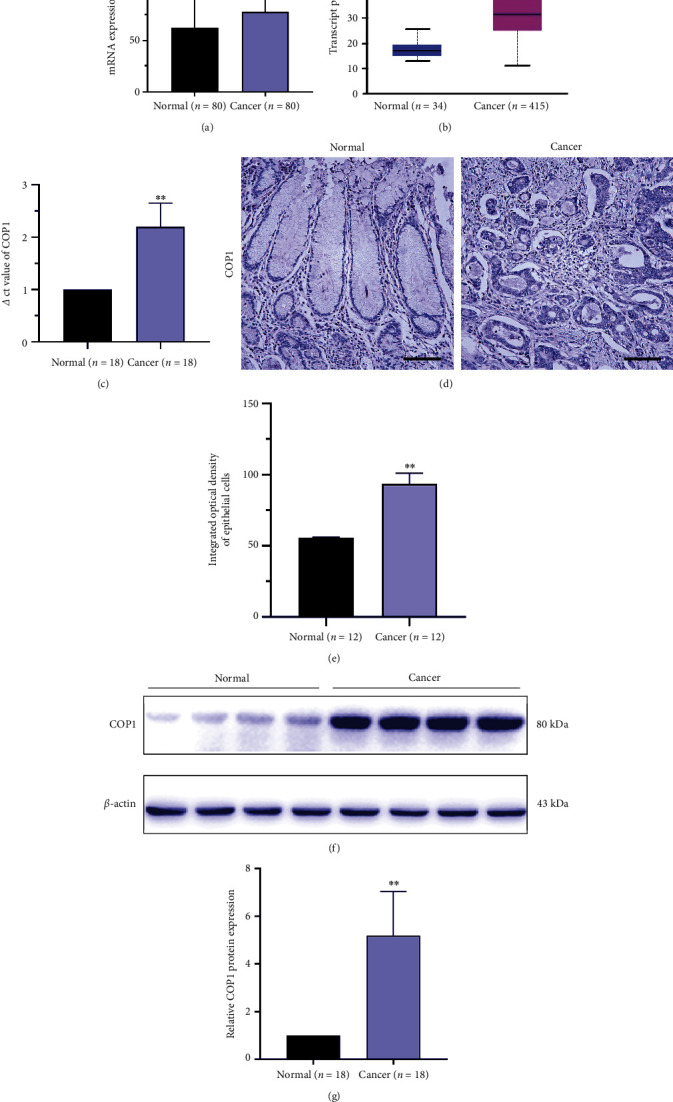
COP1 was upregulated in GC tissues. (a and b) Analysis of GEO and TCGA databases revealed over-expression of COP1 in GC tissues; (c) real-time quantitative PCR demonstrated the upregulation of mRNA expression of COP1 in GC; (d, e, f, and g) immunohistochemical staining and Western blotting analysis showed significant upregulation of the protein expression of COP1 in GC; Bar = 200 *μ*m. ∗*P* < 0.05, ∗∗*P* < 0.01, ∗∗∗*P* < 0.001.

Bioinformatics analysis demonstrated that COP1 was upregulated in GC tissues compared with the adjacent normal tissues at the level of mRNA in both GSE27342 dataset and TCGA database (Figures 1(a) and 1(b)). Moreover, the *Δ*ct values of COP1 mRNA from 18 pairs of GC tissues and the corresponding adjacent noncancerous tissues were likewise upregulated (Figure 1(c)). IHC staining and WB analysis indicated that, compared with the adjacent noncancerous tissues, the protein level of COP1 in GC was significantly upregulated (Figures 1(d), 1(e), 1(f), and 1(g)).

### 3.2. COP1 Over-Expression Precipitated the Proliferation Migration of GC Cell Lines

**Figure 2:**
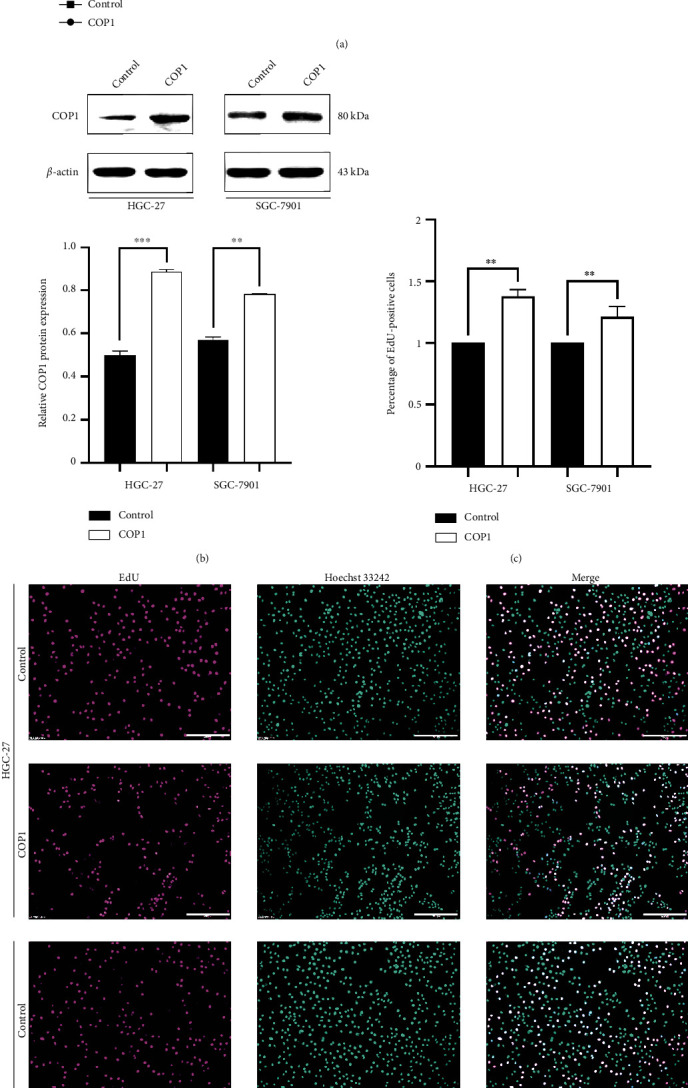
COP1 over-expression enhanced the vitality of GC cells. (a) COP1 over-expression significantly enhanced the vitality of GC cells; (b) WB assay verified the transfection efficiency of GC cell lines; (c and d) COP1 over-expression significantly increased the numbers of EdU-positive GC cells. Bar = 200 *μ*m. ∗*P* < 0.05, ∗∗*P* < 0.01, ∗∗∗*P* < 0.001.

**Figure 3:**
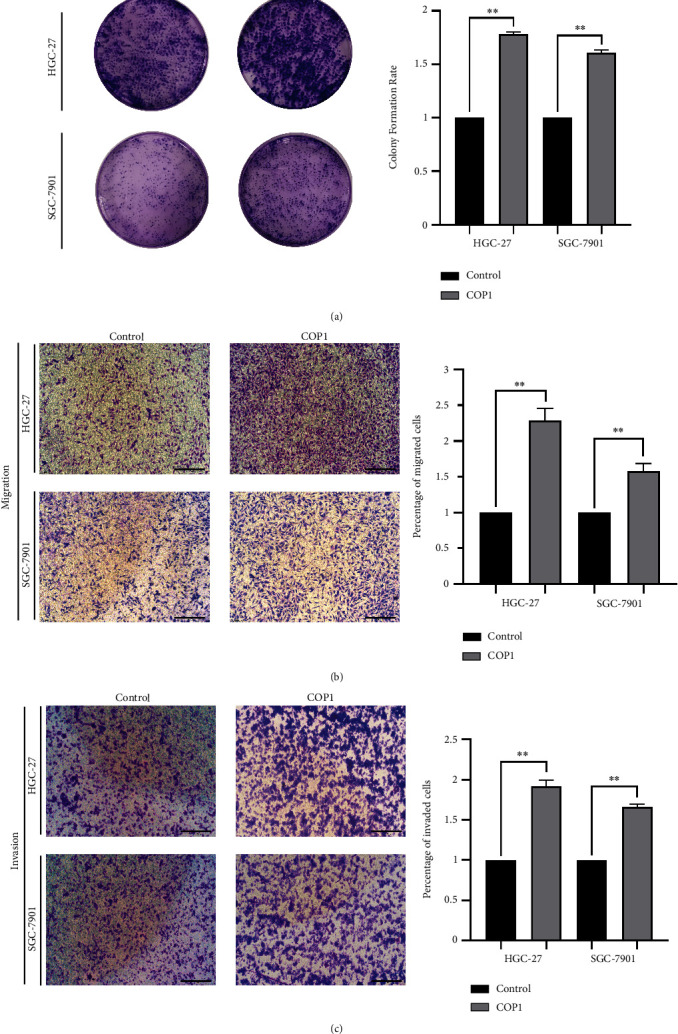
COP1 over-expression precipitated the proliferation and migration of GC cells. (a) COP1 over-expression promoted the colon formation numbers of GC cells; (b and c) COP1 over-expression significantly increased the migration and invasion of GC cells. Bar = 200 *μ*m. ∗*P* < 0.05, ∗∗*P* < 0.01, ∗∗∗*P* < 0.001.

First of all, COP1 was stably transfected into HGC-27 and SGC-7901 cell lines via lentivirus infection successfully. The induced expression profile of COP1 was verified in the above cell lines by WB (Figure 2(b)) prior to the functional experiments. CCK-8 results showed that the absorbance at 450 nm was significantly increased in COP1 over-expressed group compared to the control group from day 2 to day 5 following the infection (Figure 2(a)). The numbers of EdU-positive cells in the two cell lines were also significantly increased in the COP1 over-expressed group (Figures 2(c) and 2(d), Bar = 200 *μ*m). Similarly, compared with the control group, the numbers of cell colony formation in COP1 over-expressed group were dramatically increased in HGC-27 and SGC-7901 cell lines, respectively (Figure 3(a)). Migration and invasion assays showed that COP1 over-expression significantly multiplied HGC-27 and SGC-7901 cells (Figures 3(b) and 3(c)). These findings suggested that the over-expression of COP1 protein significantly precipitated the proliferation and migration in HGC-27 and SGC-7901 cell lines.

### 3.3. Silencing of COP1 Impaired the Proliferation and Migration of GC Cells

**Figure 4:**
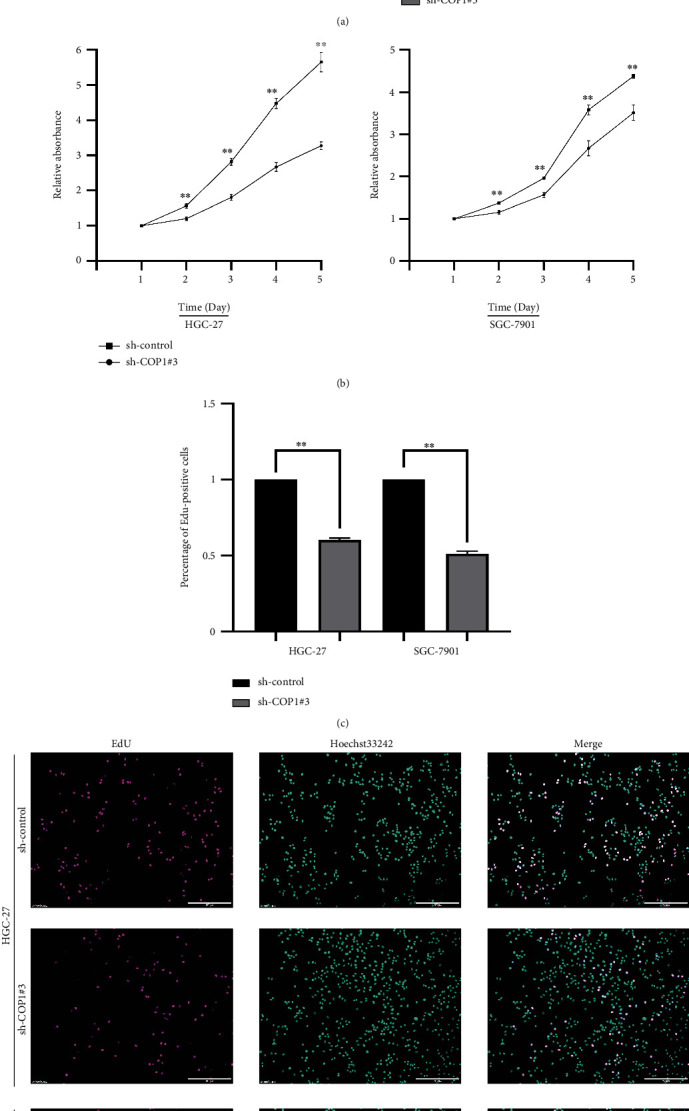
COP1 silencing reduced the vitality of GC cells. (a) WB assay verified the silencing efficiency in HEK-293T cells; (b) COP1 silencing significantly reduced the proliferation of GC cells; (c and d) COP1 silencing significantly decreased the numbers of EdU-positive GC cells. Bar = 200 *μ*m. ∗*P* < 0.05, ∗∗*P* < 0.01, ∗∗∗*P* < 0.001.

**Figure 5:**
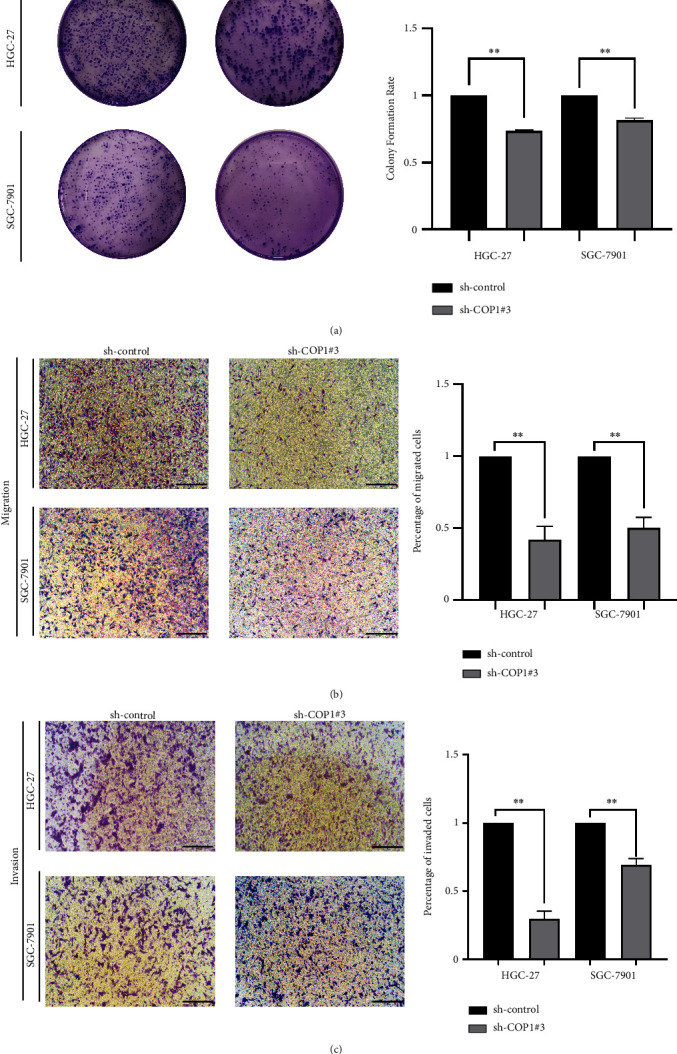
COP1 silencing inhibited the proliferation and migration of GC cells. (a) COP1 silencing inhibited the colon formation numbers of GC cells; (b and c) COP1 silencing significantly decreased the migration and invasion of GC cells. Bar = 200 *μ*m. ∗*P* < 0.05, ∗∗*P* < 0.01, ∗∗∗*P* < 0.001.

For COP1 silencing, three sequences of COP1-targeting shRNA were transiently transfected into HEK-293T cells (sh-COP1#1, sh-COP1#2, and sh-COP1#3), and the silencing efficiency of COP1 was confirmed by WB. Finally, sh-COP1#3 was designated for the optimal efficiency in silencing (Figure 4(a)). Then, the cell functional experiments were conducted for three times. CCK-8 assay revealed that the absorbance at 450 nm was significantly decreased in the sh-COP1 groups of the above two cell lines compared with their control groups (Figure 4(b)). The numbers of EdU-positive cells in each cell line were also significantly reduced in the COP1 silencing groups, respectively (Figures 4(c) and 4(d)). Likewise, compared with their control group, the numbers of cell colony formations in sh-COP1 groups were dramatically decreased (Figure 5(a)). Migration and invasion assays revealed that the silencing of COP1 significantly reduced the numbers of HGC-27 and SGC-7901 cells (Figures 5(b) and 5(c)). These data implicated that the silencing of COP1 significantly impaired the proliferation and migration of HGC-27 and SGC-7901 cells.

### 3.4. COP1 Degraded CDH18 via the Ubiquitin–Proteasome Pathway

**Figure 6:**
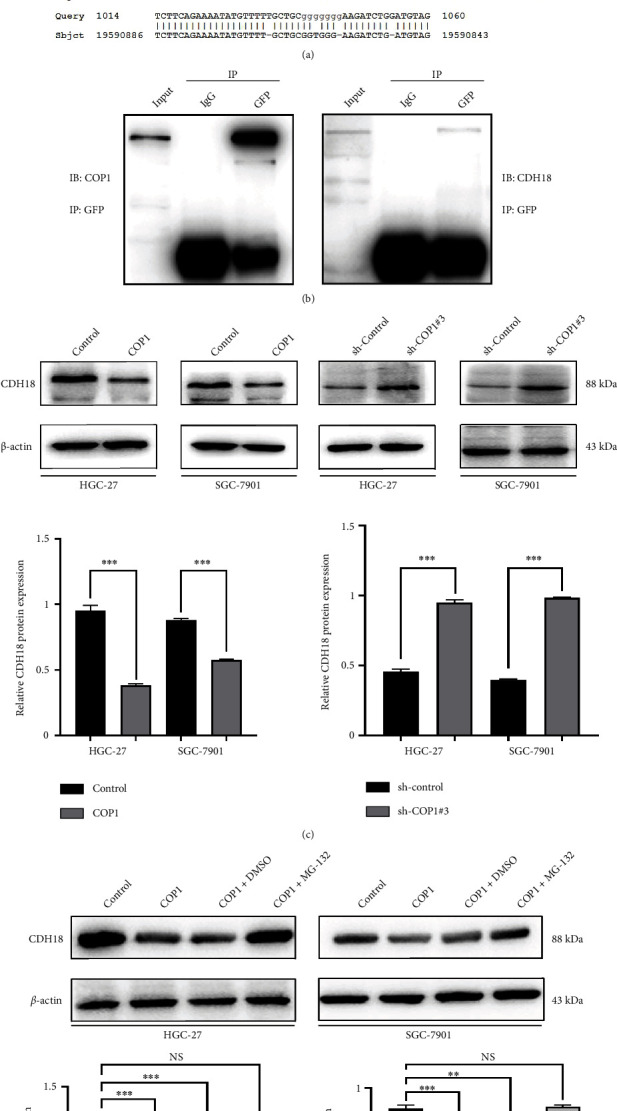
COP1 degraded CDH18 via the ubiquitin–proteasome pathway. (a) Sequence obtained from the yeast two-hybrid experiment was BLAST matched in NCBI database, and its matching degree to CDH18 reached 99%; (b) co-IP assay conducted in HEK-293T cells showed the possible bindings of COP1 and CDH18; (c and d) COP1 may degrade CDH18 via the ubiquitin–proteasome pathway. ∗*P* < 0.05, ∗∗*P* < 0.01, ∗∗∗*P* < 0.001.

We constructed a yeast plasmid bearing a GC-cDNA-library using four human GC tissues, then the yeast plasmid was hybridized with a COP1-carrying yeast plasmid. Finally, the COP1-interactive sequences were obtained from the positive yeast subsequent to yeast screen, extraction, purification, and genetic sequencing. The sequences were Basic Local Alignment Search Tool (BLAST) matched in National Center for Biotechnology Information (NCBI) database, and the CDH18 sequence exhibited high matching up to 99% (Figure 6(a)). The above result demonstrated the binding capacity of CDH18 protein to COP1, which was further confirmed by co-immunoprecipitation (co-IP) in HEK293T cells (Figure 6(b)). Moreover, WB analysis demonstrated that the over-expressing of COP1 significantly precipitated the downregulation of CDH18 in HGC-27 and SGC-7901 cells, whereas CDH18 was significantly upregulated following the silencing of COP1 (Figure 6(c)). Furthermore, COP1-mediated CDH18 degradation may be rescued by ubiquitin–proteasome inhibitor MG-132 (Figure 6(d)). These data indicated that COP1 could mediate the degradation of CDH18 with the involvement of ubiquitin–proteasome pathway.

### 3.5. The Activation of Intracellular PI3K/AKT Pathway Following the COP1-Mediated CDH18 Degradation

**Figure 7:**
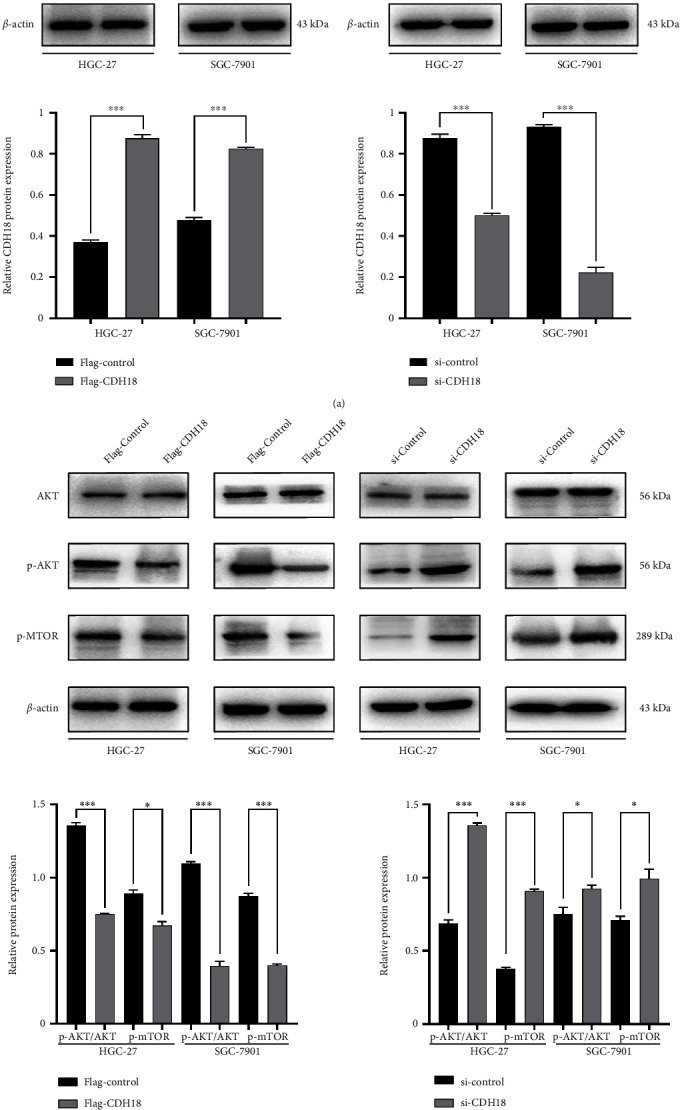
Effects of CDH18 on intracellular PI3K/AKT pathway. (a) WB verified the transfected efficiency of CDH18 in GC cell lines; (b) the effects of CDH18 on the expressions of key molecules in PI3K/AKT pathway. ∗*P* < 0.05, ∗∗*P* < 0.01, ∗∗∗*P* < 0.001.

Flag-CDH18, si-CDH18, and their corresponding blank vectors were transiently transfected into HGC-27 and SGC-7901 cell lines, respectively, with the transfecting efficiency verified by WB (Figure 7(a)). WB assay revealed that over-expression of CDH18 reduced the phosphorylation reaction of PI3K/AKT pathway, while the silencing of CDH18 reversed the decline and reached the peak (Figure 7(b)). Our findings indicated that COP1-mediated CDH18-degradation may activate the intracellular PI3K/AKT pathway.

### 3.6. Discussion

E3 ubiquitin ligase plays a vital role in the recognition of specific substrates in the UPS. On the profile of etiology, emerging evidence showed that the activity modification of E3 ligase may be the basis of human cancers [21]. Furthermore, several anti-tumor drugs targeting the members of E3-ligase-family have been tested in clinical trials [22]. COP1, a subfamily-member of E3 ubiquitin ligase, was reported to be upregulated in glioma, hepatocellular carcinoma, colorectal cancer, acute myeloid leukemia, and chronic lymphocytic leukemia, and contributed to tumor progression [14, 23–26]. In this research, the upregulation of COP1 in GC tissue was first summarized by bioinformatics, further illustrated from GC patients' tissues at both mRNA and protein levels. The in vitro data demonstrated that the over-expression of COP1 promoted the proliferation and migration of HGC-27 and SGC-7901 GC cell lines, while COP1 silencing contributed to the malignant biological behaviors of the cell lines mentioned above. Our results were consistent with the research by Li et al., who demonstrated that COP1 was upregulated in GC tissues, with p53 downregulated [27]. However, Sawada et al. [23] reported that COP1 was downregulated in GC tissues, and COP1 silencing significantly promoted the proliferation of MKN-45 GC cell line. The controversial role of COP1 in GC may be attributed to the type of p53 gene. The types of p53 in HGC-27 and SGC-7901 GC cell lines, as we chose in the experiment, were mutant, nevertheless, the p53 was wild-type in MKN-45 GC cell line. As a result, our findings were not actually conflicted with Sawada, the differences may be related to the mutation status of p53 gene in the cell line. In fact, genetic abnormalities, mutation, and inactivation of the conventional tumor suppressor genes, such as p53 and p16, may lead to high-grade transformation [28]. We don't deny the downregulation of COP1 in MKN-45 cell line in contrast to the upregulation in HGC-27 and SGC-7901 cell lines with p53-mutation, the decreased expression of COP1 may blunt the attribution of p53 as a tumor suppresser. In the present study, upregulation of COP1 was of great significance in the tumorigenesis of HGC-27 and SGC-7901 GC cells.

To elucidate the underlying mechanisms, we authenticated that CDH18 may interact with COP1 from yeast two-hybrid experiment with the utilization of GC tissues, with their interactions further confirmed by co-IP assay. Cadherin, to which CDH18 pertains, contains membrane spanning domain thus allowing for intracellular interaction with other proteins [29]. CDH18 is specifically expressed in the central nervous system, which is involved in the calcium-dependent cell-to-cell adhesion [30]. The scarcity of CDH18 was confirmed to enhance the capacity of invasion and migration in glioma cells, and the over-expression of CDH18 reduced the resistance of glioma cells to chemotherapy [30]. So far, the role of CDH18 in GC has not been reported. In this research, low-level expression of CDH18 was observed in COP1 over-expressed cell lines HGC-27 and SGC-7901. Moreover, pharmacological inhibition of the UPS by MG-132 in COP1 over-expressed GC cell lines, the decreased expression of CDH18 was turned over. The data above highly validated that CDH18 may be degraded by COP1-dependent ubiquitin-proteasome pathway. Furthermore, our data illustrated that the dysregulation of CDH18 protein resulted in significant modification of the phosphorylation level of PI3K/AKT signaling pathway. Moreover, the activation of PI3K/AKT signaling would facilitate GC cell proliferation, migration, and stemness [31]. The blockage of PI3K/AKT signaling pathway would promote GC cell apoptosis [32]. These data suggested that the reduction of CDH18 would promote GC tumorigenesis partially via the non-suppression of PI3K/AKT signaling. Together, our results demonstrated that the survival and prosperity of GC cells may be subjected to the upregulation of COP1, which degraded CDH18 via the ubiquitin–proteasome pathway thus activating the intracellular PI3K/AKT pathway.

## 4. Conclusions

COP1 over-expression precipitated the tumorigenesis of GC by degradation of CDH18 via the ubiquitin–proteasome pathway, thus activating the intracellular PI3K/AKT pathway.

## Data Availability

The data that support the findings of this study are available from the corresponding author upon reasonable request.
